# Nutritional Outcomes One Year after One Anastomosis Gastric Bypass Compared to Sleeve Gastrectomy

**DOI:** 10.3390/nu14132597

**Published:** 2022-06-23

**Authors:** Naama Shirazi, Nahum Beglaibter, Ronit Grinbaum, Wiessam Abu Ahmad, Anna Aronis

**Affiliations:** 1 Institute of Biochemistry, Food and Nutrition, Robert H. Smith Faculty of Agriculture, Food and Environment, Hebrew University of Jerusalem, Rehovot 76100, Israel; anna.aronis@gmail.com; 2 Department of Surgery, Hadassah University Hospital Mount Scopusand, the Hebrew University-Hadassah Medical School, Jerusalem 91240, Israel; bnahum@hadassah.org.il (N.B.); ronitgr@hadassah.org.il (R.G.); 3 Hadassah Braun School of Public Health and Community Medicine, Hebrew University of Jerusalem, Jerusalem 9103401, Israel; wiessam.huji@gmail.com

**Keywords:** bariatric surgeries, one anastomosis gastric bypass, sleeve gastrectomy, nutritional deficiencies

## Abstract

One Anastomosis Gastric Bypass (OAGB) and Sleeve Gastrectomy (SG) are the most common bariatric procedures performed worldwide. SG is a restrictive procedure whereas OAGB involves malabsorption as well, supposing a risk of deficiency development post OAGB. The aim of the study was to compare nutritional deficiencies and metabolic markers one year after the procedures, while adhering to the current protocols. Retrospective analysis was performed for data on 60 adults undergoing primary OAGB, compared to 60 undergoing primary SG. Mean pre-surgery BMI for SG was 42.7 kg/m^2^ and 43.3 kg/m^2^ for OAGB. A multidisciplinary team followed up with the patients at least 3 times during the first year. Mean weight loss was 39.0 kg for SG and 44.1 kg for OAGB. The OAGB group presented a significantly sharper decline in T.Chol and a trend for sharper LDL decrease; a higher increase in folate and a trend for a greater decrease in albumin and hemoglobin were observed in OAGB. For vitamin B12, D, iron and ferritin, no difference was observed between the treatment groups, although there were some in-group differences. Nutritional recommendations and adopted supplement plans minimize the risk of deficiencies and result in improvement in metabolic biomarkers one year after OAGB, which was comparable to SG.

## 1. Background

The prevalence of obesity is increasing at a high rate across all age groups, rating it as a major public health problem [1,2]. Obesity has a broad impact both on individuals who suffer from it and on the entire healthcare system; it affects financial and mental aspects and causes considerable comorbidities [3,4]. Due to the complexity of the disease and its extensive implications, a variety of attempts have been undertaken to find modern and effective treatments. The therapeutic range of these treatments is wide and varies from lifestyle modification based on a variety of diets and exercise programs, through medications and medical procedures to metabolic surgical procedures. Each treatment has a different extent of effectiveness. According to the cumulative evidence, bariatric surgery is considered to be the most effective treatment for morbid obesity [5,6]. The field of metabolic surgery is consistently evolving to develop the safest and most effective procedures, and to adapt the best complementary treatments that include nutritional and behavioral aspects. One anastomosis gastric bypass/mini gastric bypass (OAGB) and sleeve gastrectomy (SG) became common in the world, and the most common in Israel in recent years [7]. OAGB is a relatively novel surgery; therefore, the evidence on its outcomes is evolving, specifically considering the data on its complications, risks and comorbidity [8,9]. The fundamental difference in the concept of these two surgeries is that SG is a restrictive procedure, whereas OAGB involves malabsorption as well. Theoretically, the risk of developing nutritional deficiencies is increased after each of the bariatric surgeries and is even higher after OAGB due to the integration of the malabsorption component. These deficiencies supposedly cause a wide range of long-term complications, including nutritional, metabolic and neurological ones [10]. The evidence points to nutritional deficiencies or malnutrition after bariatric surgery with more severe cases after malabsorption procedures. Damage to bone density, increased anemia rates, vitamin deficiencies and nutritional adverse events have been stated as complications of malabsorptive surgeries [9,11,12]. Therefore, there is a need to monitor nutritional status as a part of the effectiveness and safety evaluation of the surgery. Despite the increasing prevalence of OAGB in Israel and worldwide, and the ongoing improvement in surgical techniques, research for appropriate nutritional care after this surgery has been lacking; therefore, OAGB patients are being advised to take nutritional supplements according to the Roux-en-Y gastric bypass (RYGB) protocol. As of now, there are no comprehensive recommendations for postoperative supplementation, nor a specific nutritional treatment protocol for the OAGB surgery that would include consideration of a significant malabsorption potential. The aim of this study was to add evidence on the nutritional outcomes and changes in biomarkers of OAGB surgery in nutritional-metabolic aspects one year after the procedure, and to compare them with the results of SG surgery.

## 2. Methods

### 2.1. Ethical Approval

This study was approved by the Helsinki Committee of the Hadassah Mount Scopus Hospital. Study approval number: 0657-18-HMO.

### 2.2. Eligibility Criteria and Power Calculation

Power calculation (80%, α = 0.05) presented a sample of N = 53 for each treatment group in order to obtain statistical significance. A sample of N = 60 was finally accepted for this study [13].

### 2.3. Study Design

A retrospective study, based on the analysis of files of OAGB and SG -patients who were admitted for surgery between the years 2017 and 2019 in the surgical department of Hadassah Mount Scopus Hospital. The registration was conducted according to the requirements of the Registrar of Bariatric Surgeries in Israel, which includes demographic data and medical information regarding the patients. From these lists, patient files were selected randomly, according to the inclusion and exclusion criteria described later in this section. After 60 suitable patient files were reached for each type of surgery, the scan of the files was completed, and the database was closed. Data were collected from the hospital patients managing software (Mahar and Ofek).

### 2.4. Inclusion Criteria

The patient files for the study included patients with no previous bariatric history who were operated on at the Hadassah Mount Scopus Hospital between 2017–2019, by the two relevant surgeries, SG and OAGB and aged 17–71.

### 2.5. Exclusion Criteria

Patients with previous bariatric operations or patient files with a large portion of missing relevant information were excluded from the study database.

### 2.6. Sources and Collecting of Information

From each patient file that was included in the database, the relevant information for the study was collected: demographic characteristics, anthropometric and blood indices before surgery and follow-up after one year. The information was generated for patient files by the medical team at the Bariatric Center during the preparation days for surgery and during postoperative follow-ups. Patients were required to attend appointments at the following time points: before the surgery, during the hospitalization process, 10 days to two weeks after the surgery, 5 weeks after the surgery, and every three months for follow-up thereafter, up to one year after the surgery. During the meetings, the patients met a multidisciplinary team and underwent several analyses. The patients were weighed by the clinical nurse and afterwards they met with a surgeon for a series of check-ups, including a patient interview, a routine physical examination, a discussion of their status and background diseases, and a profound screening of the updated blood tests. Some of the follow-ups included a meeting with a dietitian, with the aim of examining a nutritional history, blood test results for potential nutritional deficiencies and to advise on eating behavior, use of dietary supplements and exercising.

During the follow-up meetings, patients were required to present results for the following blood tests: blood count, albumin, general protein, B12, folic acid, iron, ferritin, glucose, hemoglobin A1C, lipid profile, magnesium, vitamin D, TSH and liver and kidney function. One year after surgery, PTH (Parathyroid hormone) and phosphorus tests were also required.

### 2.7. Medications

Bariatric patients at the Hadassah Mount Scopus Hospital were required to take post-surgical PPI (Esomeprazole), according to the following dosage: all patients during the first two weeks—20 mg twice a day; thereafter, SG patients were required to continue taking the drug for 3 months (20 mg per day) and OAGB patients were required to continue taking it for half a year (20 mg per day).

### 2.8. Supplements

Immediately after the surgery, the patients were advised to take supplements, as appears in Table 1, as part of routine treatment in the Hadassah Mount Scopus Hospital post-bariatric surgery. Compliance with the supplement advice was examined as a part of the interview, as mentioned previously.

### 2.9. Statistical Analysis

Comparison of quantitative variables was performed by *t*-tests for independent samples, and association with categorical variables were examined according to Chi-square tests for independent variables. To examine the differences in the research indices between the two time points and between the two treatment groups, models of repeated measures mixed design were performed where the main effect was time, and the interaction effect was time * group. The study indices for which there were baseline differences between the two study groups were analyzed by ANCOVA models to examine the differences between the two study groups while supervising the baseline indices.

## 3. Results

### 3.1. Baseline Group Characteristics

**Figure 1:**
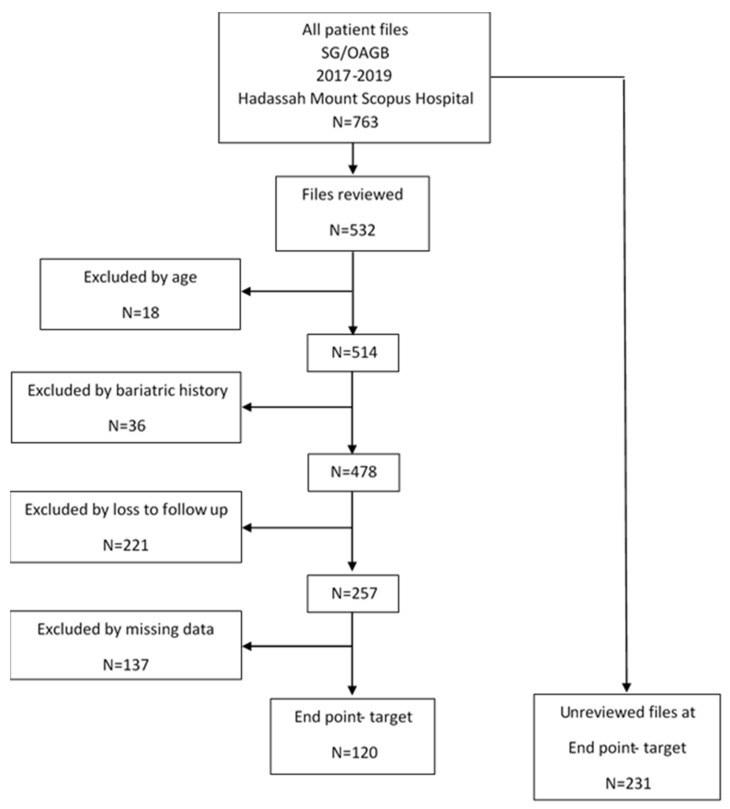
Flowchart of patient selection portfolios to create the study database.

Participant flow is presented in Figure 1.

A trend for a younger age was observed in the sleeve gastrectomy group. All background data, gender composition, age, height, weight and BMI before surgery in both groups were examined by Levene’s test for equality of variances, which showed that there is no significant difference in all parameters; therefore, the groups could be considered as statistically equal (Table 2).

The mean weight loss was 39.0 (+/−10.0) kg for SG patients and 44.1 (+/−16.0) kg for OAGB patients. Post-operative changes in BMI and weight are presented in Table 3. As expected, the analysis exposed trends for more effective TWL and BMI losses, as well as for lower absolute weight and BMI values for OAGB surgery than for SG.

### 3.2. Weight and Malnutrition Indices

#### 3.2.1. Weight-Related Anthropometric Measurements

At T1, one year after the surgery, there is a borderline significance for slightly lower weight for OAGB, as well as a trend for slightly higher BMI for SG groups. Although these absolute parameters do not reflect the progress after the surgery, total weight loss (TWL) is the leading index today for comparison of weight loss after bariatric studies. TWL in kg showed a borderline significance for a greater weight loss after OAGB compared to SG. BMI loss demonstrated a similar trend, supporting that OAGB has a potential for a more significant weight loss one year after surgery (Table 3).

#### 3.2.2. Albumin as Malnutrition and Prognostic Parameter

A decrease in albumin was observed for both procedures compared to the baseline. It is important to note that the albumin after both surgeries remained within the normal range, and no significance was observed between the OAGB and SG groups (Table 4).

### 3.3. Metabolic Indices

In order to compare the potential of the two surgeries in improving comorbidities and metabolic status of patients, blood indices, biomarkers of the metabolic syndrome and comorbidities related to obesity were analyzed.

#### 3.3.1. Lipid Profile

The parameter in which the improvement was more prominent under the OAGB is the total cholesterol, compared to SG (*p* = 0.044). In addition, the OAGB group showed a trend of significant improvement in LDL when compared with SG. For both surgeries, an in-group improvement in LDL was observed. For triglycerides and HDL, an improvement was obtained one year after both surgeries, without statistical significance between the surgery groups (Table 4).

#### 3.3.2. Hemoglobin A1c

Hemoglobin A1C was one of the three indices with unequal variances (together with iron and B12 vitamin), as found by the Levene’s test; therefore, ANCOVA analysis was performed. No significant difference was observed in A1C between the SG and OAGB one year after surgery. The Hg A1C improved in both groups regardless of the type of treatment (Table 5).

### 3.4. Micronutrients

In order to detect increased risk for nutritional deficiencies that may lead to long-term complications, micronutrient indices were analyzed. Blood levels of minerals and vitamins, or their biochemical markers, were selected according to the supplement regimens and follow-up in bariatric surgeries.

#### 3.4.1. Iron Status

ANCOVA analysis demonstrated no change in iron levels between the groups (Table 5). However, post hoc analyses showed an increase in iron level when comparing mean levels before and after surgery in each group.

Hemoglobin decreased significantly in both surgeries, with a more intensive decrease for OAGB. Despite the decline, after one year the hemoglobin levels remained within the normal range (Table 4). In contrast, changes observed in ferritin levels over time were neither significant, nor affected by the type of treatment (Table 4).

#### 3.4.2. Bone Health

The main indices for bone health are vitamin D, calcium and PTH. They indicate a risk for lack of bone tissue and are important for preservation of normal metabolism of bones after bariatric surgeries.

An improvement in vitamin D levels was observed for both groups regardless of the type of surgery. This increase is likely due to postoperative supplementation (Table 4).

Calcium levels showed a mixed trend. In SG, an increase in calcium level was observed, whereas in OAGB a slight decrease appeared. A pairwise comparison analysis revealed a significant increase of 0.266 mg/dL in calcium one year after the SG procedure, whereas for OAGB there was no change.

Due to an unequivocal requirement for monitoring PTH level, the data collection for PTH was unsuccessful.

#### 3.4.3. Vitamins B12, B9

B vitamins are recommended supplements after most bariatric surgeries. ANCOVA test was performed for vitamin B12 due to the variance between the groups at time zero. The result obtained at T1 demonstrated a trend (*p* = 0.054) for higher levels of B12 for OAGB (Table 5). A significant increase was observed for folate levels after both surgeries, with significantly higher levels after OAGB (*p* = 0.022) (Table 4).

## 4. Discussion

In this study, we compare one-year post-surgery outcomes of two leading bariatric surgeries in Israel and in other countries. The aim was to examine whether OAGB results in significant outcomes beyond better weight loss and affects nutritional and metabolic risk factors. Shohar et al., showed that RYGB malabsorptive surgery creates a more significant weight loss compared to restrictive SG surgery due to the different surgical techniques [14]. In the current study, a trend was demonstrated by two weight loss indices, TWL and BMI loss, which indicated a greater weight loss one year after OAGB compared with SG.

Albumin is a prognostic measure for protein-calorie malnutrition; therefore, it is monitored before and after bariatric surgery [15]. Its level decreased a year after both surgeries compared to the baseline, but remained within the normal range, i.e., did not indicate a risk of malnutrition or a poor post-operative prognosis. Albumin levels can be masked by various factors, such as decreased fluid volume in the body and adaptation, particularly decreased protein catabolism. However, in severe cases of malnutrition, a decrease in albumin below the normal range can be observed also after bariatric surgeries, which can prompt redo surgeries [16].

Bariatric surgery is known to result in a marked improvement in obesity complications and comorbidity [17], such as remission in diabetes, reduction in risk of heart disease and improvement in dyslipidemia [18,19,20,21]. The present study examines the question of whether one of the procedures has an advantage in improving metabolic biomarkers that indicate obesity-associated comorbidity. In this context, changes in lipid profiles were compared. It can be assumed that OAGB surgery has a higher potential for improvement in the patient’s lipid profile because this surgery incorporates significant malabsorption. In practice, an improvement in lipid profile indices was observed for both surgeries one year postoperatively, with a between-group significance observed for total cholesterol after OAGB surgery, and a trend LDL reduction.

Metabolic changes also concluded in hemoglobin A1C decline one year after the surgery. A variance between the groups at T0 may reflect the tendency or bias in decision making to perform OAGB surgery in more severe cases of unbalanced diabetes. One year after the surgery, no significant advantage for a particular treatment was observed.

The discussion on the supplementation regime after OAGB remains open due to poor relevant data. One of the principal issues of the current study was whether the malabsorptive component results in more significant nutritional deficiencies one year after OAGB compared to SG. The data was collected regarding the main nutrients that are routinely followed up post-surgically.

Iron status is commonly disrupted among obese individuals [22], and, according to the evidence, depletes after bariatric surgery [23]. A high incidence of anemia appears post-operatively due to physiological changes, disruptions in absorption, insufficient intake of iron-rich food, as well as intolerance to meat consumption [24]. Different types of alimentary anemia are being diagnosed according to hemoglobin levels, RBC count and volume, and the cause of decrease in hemoglobin [25]. A decrease in hemoglobin levels was observed after both surgeries, with a trend to a greater reduction after OAGB. A comprehensive review article presents that for a malabsorptive RYGB surgery, the incidence of iron deficiency anemia is higher than after restrictive SG [26]. In the current study, an improvement in iron levels was observed a year after the surgery, probably due to proper nutritional supplementation for the patients, and regardless of the type of the procedure they underwent. This is a desirable nutritional result in bariatric patients, indicating that the postoperative supplementation is consistent with the procedure and gaping the changes that iron status undergoes post-operatively. It is important to note that the results include a follow-up of only one year post-operatively, and longer follow-up is needed to draw conclusions about the dynamics of change in the iron status in these two surgeries. The decrease in hemoglobin presented in this study may continue below the normal range, but the current data are insufficient to conclude and predict the further drift.

The patients in the study showed normal levels of vitamin B12 and folate preoperatively. A year after, a significant increase in folate was observed in both groups, whereas vitamin B12 did not change. Therefore, according to the results, patients who had prior normal levels of B vitamins can use supplementation according to the protocol used for this study. It should be noted that some patients start at low levels and may require closer monitoring to prevent the deficiency.

The mechanisms of the effect of bariatric surgery on possible damage to bone health are not completely understood. Weight reduction (including muscle mass), decrease in mechanical pressure on bones and nutritional components probably constitute a part of this complex process. The nutritional aspect of bone health is affected by decreased levels of calcium and vitamin D parallel to increased levels of PTH; therefore, it is important to measure their levels before and in long-term post-surgical follow-up as a predictor of bone damage. From these follow-ups, treatment options may arise by appropriate supplementation and thus prevention of bone damage [27,28].

Levels of vitamin D improved after both surgeries regardless of the type of procedure. Intake of vitamin D in bariatric patients’ diets is not expected to reach RDA, so it can be assumed that the improved levels of the vitamin as a result of the adherence to the recommended supplementation. In addition, a decrease in fat mass reduces the storage of the non-functional vitamin D [29]. To present a complete picture of bone-related changes in the post-surgery period, vitamin D and calcium should be interactively analyzed with PTH levels. However, PTH was not consequently followed up, which complicates the discussion on decrease in calcium levels in OAGB, and its increase in SG patients. In both groups, calcium remained within the normal ranges, whereas the changes may be associated with compliance with the supplementation plan, but also could reflect PTH variability. In order to better monitor bone health and assess the risk of bone damage, the recommendations which include DEXA test before surgery and two years after should be completed, as well as routine follow-up of PTH levels every six months [30,31].

The present study sheds light on the importance of multidisciplinary team care in the perioperative and in the short term after surgery. It demonstrates that proper treatment, combined with nutrition consulting, using an existing diet protocol and supplementation regimen can protect from significant malnutrition complications.

The research has some limitations. Thus, the study included only patients who visited for follow-up, i.e., a population with high compliance to treatment. It should be noted that according to Israel Center for Disease Control [7], about 50% of bariatric patients did not visit a nutritionist even once after surgeries in the period of 2013–2020. Poor compliance to the supplement treatment may be observed among those who dropped out from the follow up. Moreover, among those who do visit a nutritionist, many bariatric patients in Israel continue follow-up and nutritional treatment at HMOs and not in hospitals and were therefore absent and not sampled in the study. Lack of data on PTH and DEXA tests also presented a limitation for appropriate bone health analysis. COVID-19 epidemic could bias the study population. Restrictions in elective visits to hospitals disrupted a regular follow-up plan, reducing patient’s follow-up visits to hospital clinics due to fear of infection.

Optimal outcomes of bariatric surgeries are durable depend on lifestyle modification, dietary changes, and proper use of nutritional supplements. It has a crucial impact on the overall health of the patient over the years. Therefore, studies including longer follow ups are needed. It is worthwhile to include information about patients undergoing follow-up at HMOs, compliance to menus and dietary supplements, physical activity and even conduct prospective research that will combine a detailed eating record. Efficacy and nutritional status of all the variety of bariatric surgeries remain of great interest for investigation.

## 5. Conclusions

Our research demonstrated again that bariatric surgery is an effective treatment for weight loss and improvement of comorbidities. Neither significant nutritional deficiencies were observed after OAGB, nor any better metabolic improvement compared to SG in short-term 1-year follow up. The current treatment protocol for RYGB, including follow-ups by a multi-disciplinary medical staff and nutritional supplementation can be appropriate for OAGB postoperative care. It is recommended to ensure long-term follow-up of albumin and to adhere to dietary supplement of iron, folate and vitamin B12 after OAGB. The data collected in the current study was insufficient for drawing conclusions regarding bone metabolism, emphasizing the need for further research.

## Figures and Tables

**Table 1:** Clinical guidelines for dietary supplements after bariatric surgery at Hadassah Hospital on Mount Scopus.

Type of Dietary Supplement	OAGB	SG
Calcium mg/day	500 × 2	500
Multivitamin for children (chewable) pills/day	4	4
Whitman D IU/day	According to blood test/3000	According to blood test/3000
Liquid iron mg/day	50	-
Complex B/B12	According to blood test	According to blood test

**Table 2:** Pre-operative study’s groups characteristics at baseline.

Variables	SG		OAGB		*p*
Gender	N (%)		N (%)		
Man	20 (47.6%)		22 (52.4%)		0.702
Woman	40 (51.3%)		38 (48.7%)		
	N	M (SD)	N	M (SD)	*p*
Age (Y)	60	39.7 (15.5)	60	44.0 (11.0)	0.078
Weight (kg)	59	117.4 (21.3)	60	121.3 (24.0)	0.352
Height (m)	58	1.65 (0.1)	60	1.67 (0.1)	0.363
BMI (kg/m^2^)	58	42.7 (4.7)	60	43.3 (6.7)	0.595

**Table 3:** Weight and BMI trends one year after surgery, T1 time.

	SG		OAGB		*p*
Variables	N	M (SD)	N	M (SD)	
Weight T1(kg)	55	78.2 (18.2)	51	79.2 (16.0)	0.051
BMI T1 (kg/m^2^)	54	28.3 (4.7)	51	28.2 (5.0)	0.091
TWL (kg)	55	39.0 (10.0)	51	44.1 (16.0)	0.051
WL (%)	55	33.5 (7.0)	51	35.4 (8.0)	0.199
EWL (kg)	59	48.5 (16.6)	59	51.1 (20.2)	0.388
EWL (%)	54	84.9 (21.7)	51	87.6 (24.0)	0.545
BMI loss (kg/m^2^)	54	14.2 (3.4)	51	15.5 (5.0)	0.091
BMI loss (%)	54	33.5 (7.1)	51	35.4 (8.0)	0.207

**Table 4:** Comparison of outcomes between the two groups, one year after surgery.

	SG			OAGB			MAIN EFFECT (TIME)		INTERACTION (TIME*TREATMENT)	
Variables	N	M (SD) T0	M(SD) T1	N	M(SD) T0	M(SD) T1	F	*p*	F	*p*
HB gr/dL	57	14.0 (1.3)	13.5 (1.4)	56	14.0 (1.3)	13.0 (1.6)	(1, 111) = 46.6	<0.001	(1, 111) = 7.0	0.009
Ferritin ng/mL	40	67.7 (54.0)	76.4 (64.8)	34	86.0 (60.3)	80.2 (71.1)	(1, 72) = 0.044	0.835	(1, 72) = 1.072	0.304
Vitamin D ng/m	40	23.9 (17.0)	44.5 (32.7)	40	21.1 (11.8)	38.2 (37.3)	(1, 78) = 23.0	<0.001	(1, 78) = 130.0	0.648
Albumin gr/dL	42	42.8 (3.2)	41.9 (3.3)	45	42.4(3.6)	40.2(3.3)	(1, 85) = 16.0	<0.001	(1, 85) = 3.0	0.088
Folate ng/mL	38	11.4 (11.3)	14.4(9.9)	36	9.9 (6.2)	19.1 (13.2)	(1, 72) = 18.0	<0.001	(1, 72) = 5.5	0.022
Calcium mg/dL	36	9.3 (0.4)	9.6 (0.4)	35	9.4 (0.6)	9.3 (0.5)	(1, 69) = 4.521	0.037	(1, 69) = 10.0	0.002
T.Chol mg/dL	60	187.1 (35.8)	173.8 (30.3)	58	181.5 (37.5)	155.4 (35.3)	(1, 115) = 39.3	<0.001	(1, 115) = 4.164	0.044
LDL mg/dL	56	113.3 (29.2)	103.0 (26.9)	53	111.2 (33.9)	90.4 (30.4)	(1, 107) = 29.3	<0.001	(1, 107) = 3.4	0.07
HDL mg/dL	54	44.9 (9.1)	52.3 (8.9)	50	43.8 (12.1)	49.4 (12.7)	(1, 102) = 35.9	<0.001	(1, 102) = 0.8	0.39
TG mg/dL	59	161.9 (98.0)	88.3 (31.5)	58	164.9 (89.5)	90.3 (30.9)	(1, 115) = 93.8	<0.001	(1, 115) = 0.004	0.95

**Table 5:** Parameters with unequal variables analyzed by ANCOVA at T1 time point.

			SG				OABG		
Variables	Mean T0 (SD)	Mean T1 (SD)	Mean Adjective *	N	Mean T0 (SD)	Mean T1 (SD)	Mean Adjective *	N	*p* **
Iron mg/dL	77.7 (26.2)	88.9 (47.9)	88	49	60.8 (18.5)	76.5 (37.0)	77.4	46	0.246
B12 ng/mL	327.2 (112.2)	365.5 (159.1)	371.1	51	430.7 (171.3)	462.1 (237.6)	455.9	46	0.054
Hb A1C%	5.6 (0.9)	5.2 (0.5)	5.3	33	6.3 (1.1)	5.2 (0.7)	5.1	38	0.127

* Mean adjective is related to ANCONA test at T1 point. ** *p* related to between-group analysis.
